# Voxel-based identification of local recurrence sub-regions from pre-treatment PET/CT for locally advanced head and neck cancers

**DOI:** 10.1186/s13550-019-0556-z

**Published:** 2019-09-18

**Authors:** J. Beaumont, O. Acosta, A. Devillers, X. Palard-Novello, E. Chajon, R. de Crevoisier, J. Castelli

**Affiliations:** 1 Univ Rennes, CLCC Eugène Marquis, INSERM, LTSI - UMR 1099, 35000 Rennes, France; 2 Department of Radiotherapy, Centre Eugene Marquis, avenue de la Bataille Flandre Dunkerque, 35000 Rennes, France

**Keywords:** Radiotherapy, Locally advanced head and neck cancers, Recurrence prediction, Radiomics, Voxel-based analysis

## Abstract

**Background:**

Overall, 40% of patients with a locally advanced head and neck cancer (LAHNC) treated by chemoradiotherapy (CRT) present local recurrence within 2 years after the treatment. The aims of this study were to characterize voxel-wise the sub-regions where tumor recurrence appear and to predict their location from pre-treatment ^18^F-fluorodeoxyglucose (FDG) positron emission tomography (PET) images.

**Materials and methods:**

Twenty-six patients with local failure after treatment were included in this study. Local recurrence volume was identified by co-registering pre-treatment and recurrent PET/CT images using a customized rigid registration algorithm. A large set of voxel-wise features were extracted from pre-treatment PET to train a random forest model allowing to predict local recurrence at the voxel level.

**Results:**

Out of 26 expert-assessed registrations, 15 provided enough accuracy to identify recurrence volumes and were included for further analysis. Recurrence volume represented on average 23% of the initial tumor volume. The MTV with a threshold of 50% of SUVmax plus a 3D margin of 10 mm covered on average 89.8% of the recurrence and 96.9% of the initial tumor. SUV and MTV alone were not sufficient to identify the area of recurrence. Using a random forest model, 15 parameters, combining radiomics and spatial location, were identified, allowing to predict the recurrence sub-regions with a median area under the receiver operating curve of 0.71 (range 0.14–0.91).

**Conclusion:**

As opposed to regional comparisons which do not bring enough evidence for accurate prediction of recurrence volume, a voxel-wise analysis of FDG-uptake features suggested a potential to predict recurrence with enough accuracy to consider tailoring CRT by dose escalation within likely radioresistant regions.

## Introduction

Chemoradiotherapy (CRT) is a standard treatment for non-resected or unresectable locally advanced head and neck cancers (LAHNC) [1–3]. Radiotherapy (RT) combined with cetuximab has been established as a potential alternative standard treatment, especially useful when concomitant chemotherapy cannot be applied [4]. Overall survival of these patients is highly correlated with the loco-regional recurrence, which arises in 20% to 40% of cases [5–9].

Dose escalation within the tumor may increase local control, although limited by the risk of toxicities [10, 11]. Positron emission tomography (PET) with ^18^F-fluorodeoxyglucose (FDG) can play a major role in guiding local dose escalation allowing to determine regions prone to be radioresistant and thus can be likely at the origin of the recurrence [12, 13]. Thus, the prescribed dose can be homogeneously delivered within the 18-FDG-subvolume (dose painting by contour) [14] or heterogeneously planned thereby appearing as a function of the signal intensity at each voxel in the biologic image (dose painting by numbers) [15]. However, recent studies which assessed the outcomes of dose painting in head and neck cancers suggested that there is no strong evidence of a correlation between recurrence and the standard uptake value (SUV) [14–21]. Other volumetric PET parameters such as metabolic tumor volume (MTV) and total lesion glycolysis (TLG) have been correlated with overall survival and local control [22, 23]. Particularly, the MTV may be useful in the identification of a global recurrence volume.

A recent study, based on 42 patients with a local failure after a radiotherapy treatment, showed that the MTV computed with a threshold of 50% of the maximum SUV of the tumor plus a margin of 10 mm (MTV_50 + 10_) covers the majority of the recurrence [24]. This volume closely corresponds to the gross tumor volume (GTV), limiting the use of dose escalation strategies. Chaput et al. and Legot et al. [25, 26] also investigated the location based on a spatial alignment of the MTV with the recurrence region suggesting that hypermetabolic sub-regions are likely within the relapse volume. Nevertheless, MTV is a rather global feature which does not allow to individually identify voxels or local sub-regions likely responsible for the relapse. There is nowadays a lack of fine characterization of local recurrence at a voxel level able to accurately predict the sub-regions likely responsible of recurrence thus enabling the tailoring of the prescribed dose. This problem is not solved partly because in head and neck, the identification of common regions in both pre- and post-treatment images via registration is not an easy task. Indeed, important anatomical modifications may arise between both time points due not only to weight loss and tumor regression but also to the different anatomic position and the presence of image artifacts. Characterization of PET images may go beyond simple voxel-wise SUV values, but rather including other radiomics-like [27–29] features, which may capture both global and local FDG-PET uptake characteristics. Radiomics features may help unraveling the underlying structure of the tumor and therefore add fundamental quantitative information to the characterization of those voxels. The advantage is that each voxel is characterized by a set of hand-crafted features encompassing multiscale information.

In this context, the aims of this study were (i) to identify the volume of tumor recurrence, (ii) to characterize it in terms of global and local features, and (iii) to build a voxel-wise machine learning model able to predict, from pre-treatment PET images, sub-regions where recurrence is likely to occur.

## Materials and Methods

### Inclusion criteria

All consecutive patients treated in our center with definitive concurrent CRT or RT and cetuximab for LAHNC between January 2012 and December 2015 were retrospectively reviewed. Inclusion criteria were an age between 18 and 75 years, T3-4 or N+ stage, no surgery before RT, no history of cancer, a PET/CT performed within 8 weeks prior to the start of RT, no metastasis at diagnosis, a minimal follow up of 6 months, and a local recurrence confirmed by PET/CT as primary event.

### Patients and treatment characteristics

This retrospective study enrolled 26 patients. All patients underwent intensity modulated radiotherapy (IMRT) using a total dose of 70 Gy (2 Gy/fraction/day, 35 fractions), with simultaneous integrated boost technique [30] and concomitant chemotherapy or cetuximab [20] if the patients were not fit for chemotherapy. The protocol for planning and treatment was the same as previously described [21].

Physical evaluation and laryngoscopy were performed after RT every 3 months for the first 2 years and then every 6 months thereafter. A PET/CT was systematically performed between 3 and 6 months after treatment. During follow up, a PET/CT was also performed for patients with clinical recurrence.

Median time to recurrence after treatment was 4 months (ranging from 3 to 22 months).

### PET/CT acquisition

All patients underwent FDG PET/CT for staging before treatment. The patient lasted at least 4 h prior to the injection of 4 MBq/kg of ^18^F-FDG. If not contraindicated, intravenous contrast agents were administered before CT scanning. After a 60-min uptake period of rest, patients were imaged with a PET/CT imaging system Discovery ST (General Electric Medical Systems; General Electric Healthcare, Milwaukee, Wis). First, a CT (120 kV, 80 mA, 0.8-s rotation time, slice thickness 3.75 mm) was acquired. A PET scan was performed immediately after the acquisition of the CT. Images were acquired from the base of the skull to the midthigh (3 min/bed position). PET images were reconstructed by using an ordered-subset expectation maximization iterative reconstruction (two iterations, 28 subsets) and an iterative fully three-dimensional image. CT data were used for attenuation calculation. The same protocol was used for follow-up. The study was approved by the institutional review board.

### Recurrence volume identification

**Fig. 1:**
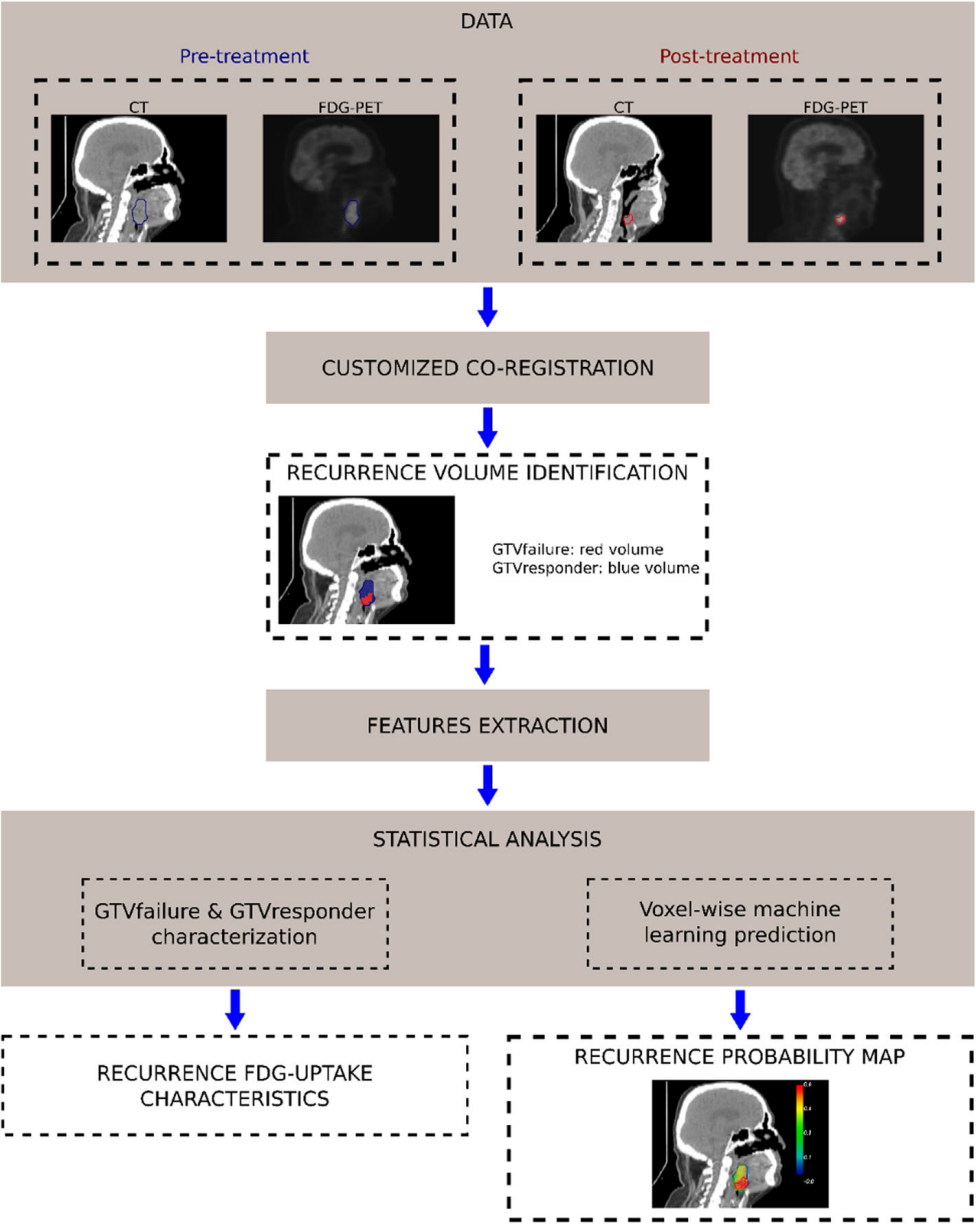
Workflow of the study. (i) Intra-individual registration of post-treatment data to the pre-treatment space allows the definition of both the region of recurrence volume (GTVfailure) and the responder volume (GTVresponder). This step is followed by (ii) a regional analysis allowing to characterize the recurrence and (iii) a voxel-wise analysis (feature extraction and classification) which yields a probability map of recurrence

The primary tumor was segmented on both pre-treatment and recurrence PET/CT. A rigid co-registration step was then performed between pre-treatment and recurrence PET/CT (Fig. 1) using a customized method based on common structures. After co-registration, two different sub-regions were labeled: (i) the recurrence volume (GTVfailure), defined as the anatomical region where the pre-treatment and the recurrent primary tumors intersected; (ii) the responder volume (GTVresponder), defined as the anatomical region where no overlap existed between the pre-treatment tumor and the recurrent tumor.

**Fig. 2:**
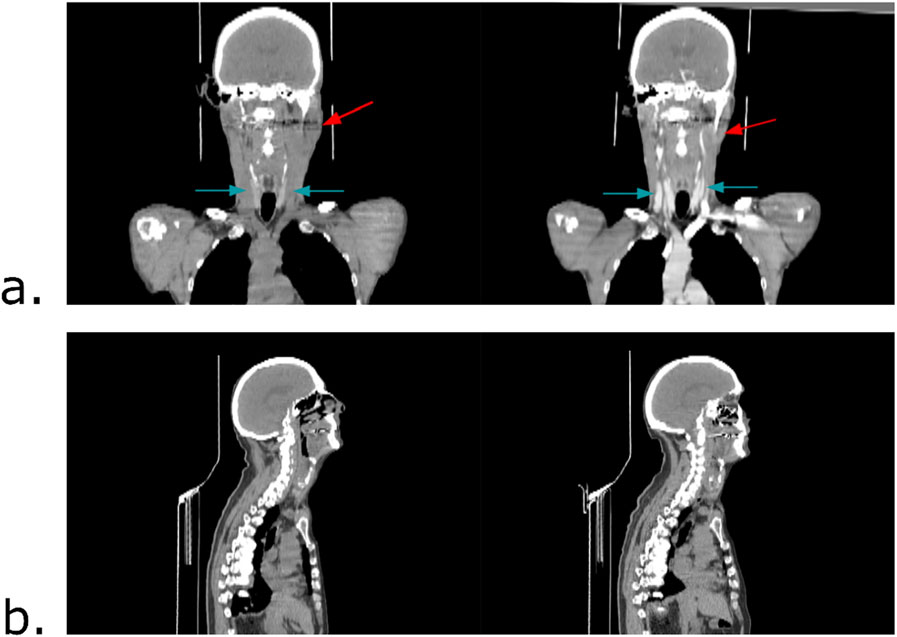
Intra-individual differences that can be found in CT images acquired at different time points. Left: pre-treatment CT. Right: post-treatment CT. **a** Red arrow: changes of patient anatomy after the treatment. Blue arrow: intensity differences due to the injection of a contrast agent in the post-treatment CT (right). **b** Differences in the position of the patient between pre- and post-treatment CT. Here, the inclination of the head has changed

The customized rigid registration method was based on a block matching algorithm [31, 32] devised to address the issues arising when registering head and neck anatomies between two endpoints after radiotherapy. Specifically, the weight loss and tumor regression induced by the chemo-radiotherapy treatment may lead to important changes in patients’ anatomies, as shown in Fig. 2. Patient positions and differences in contrast agents can also hamper the registration process. To account for these anatomical changes, the registration was performed on distance maps computed from bony structures [31]. Thus, bone segmentations were firstly obtained by thresholding CT images with a value of 200 HU followed by an Euclidean distance map computation [33]. This step was followed by a finer registration of bony structures within manually selected ROIs encompassing the pre-treatment tumor.

The matching of the bony structures was first quantitatively assessed using the dice score but eventually the registrations were qualitatively assessed by a senior radiation oncologist. Patients with inaccurate visual registration of the tumor area were excluded from the study. In a second time, four physicians also reviewed the registrations. Inter-rater reliability was evaluated using Fleiss’s Kappa method.

### Features extraction

FDG-uptake features were extracted from pre-treatment PET images at different spatial scales. A first regional approach was used to globally characterize both GTVfailure and GTVresponder with different thresholds, while a voxel-wise analysis was performed to characterize local properties of these regions. These features were then used to train a model able to predict the GTVfailure volume as depicted in Fig. 1.

#### Features from recurrence and responder volumes

Histogram features were extracted from pre-treatment PET images for both GTVresponder and GTVfailure regions as presented in Table 1.

**Table 1:**
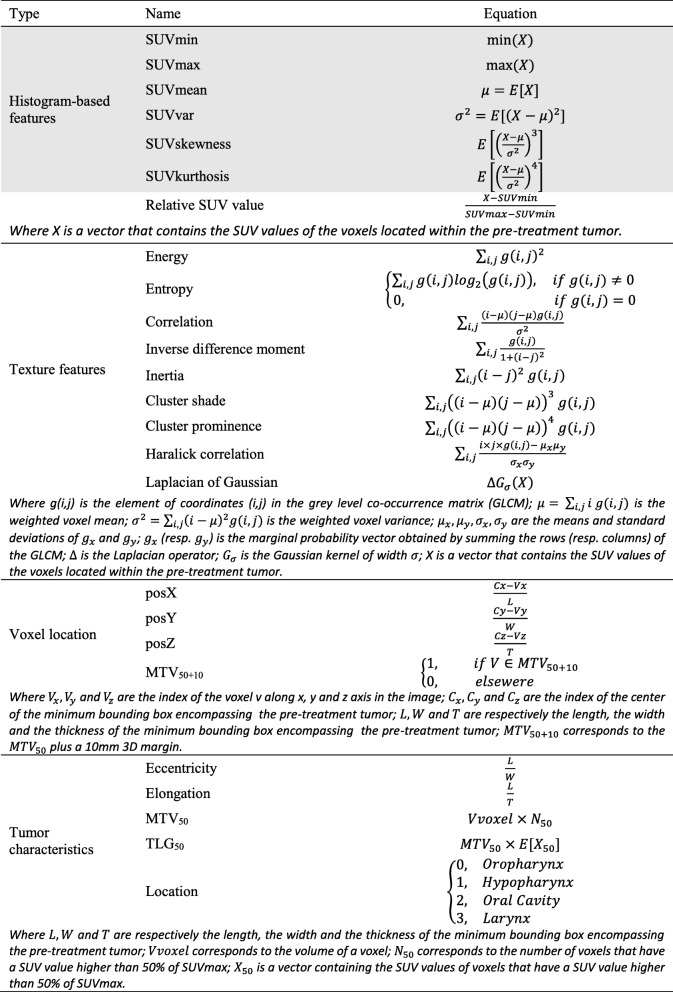
Equations of radiomics features used to characterize the recurrence volume (grey background) and to train the random forest

Based on pre-treatment PET, the MTV of the primary tumor was computed with thresholds ranging from 0 to 100% (step − size = 1 % ) of the maximum SUV (SUVmax). The coverage of both GTVfailure and GTVresponder by the MTV was then computed for each threshold.

#### Voxel-wise analysis

The primary tumor was characterized at the voxel level by different geometric and image features [34]. Thus, for each voxel *v*, the features extracted were the relative SUV value of *v*; the Euclidean distance [33] between *v* and *vMax*, with *vMax* the voxel with the highest averaged SUV value (computed within the 26 neighborhood); the Euclidean distance between ***v*** and *vMin*, with *vMin* the voxel with the lowest averaged SUV value (computed within the 26 neighborhood); the Euclidean distance between *v* and *vSurf*, with *vSurf* the closest voxel within the primary tumor surface; Haralick texture features [35], computed within a neighborhood of two voxels; the Laplacian of Gaussian (LoG) value of *v*; the location of the voxel *v* within the pre-treatment tumor; the inclusion of the voxel *v* within the MTV_50+10_ volume; and the overall tumor characteristics: 3D shape descriptors, MTV_50_, TLG_50_, and tumor location (oropharynx, hypopharynx, …). In order to deal with rotation invariant texture features, PET images were resampled to isotropic voxel size. Haralick texture features were computed on normalized grey level co-occurrence matrix with 64 bins. Those features were implemented as presented Table 1, thereby obtaining 22 features per voxel in total.

### Statistical analysis

#### Recurrence and responder volume characterization

Wilcoxon signed-rank test [36] was used to assess whether global histogram features computed within GTVfailure were significantly different (*p* value ≤ 0.05) from those computed within GTVresponder.

#### Voxel-wise prediction of the recurrence volume

A random forest (RF) model [37, 38] was trained with voxel-wise features. The probability of belonging to the GTVfailure for each voxel was computed as the supervised output by dividing the number of trees which predicted that a voxel belonged to GTVfailure by the total number of trees of the model.

The RF was trained using ranger [39], a C++ software tool that implements the probability random forest algorithm [38]. The training was performed using a leave-one-out cross-validation framework with ranger default parameters, excepted for the number of trees, which was set to 10,000.

The mean out-of-bag (OOB) prediction error [40] was computed to assess the performance of the model on the training data set, while the area under the receiver operating curve (AUC) was used to assess these performances on the testing data. It should be noted that the relation between the OOB prediction error and the AUC is *OOB* = 1 − *AUC*.

The Mean Decrease Gini Index (MDGI), yielded by ranger, allowed to assess the importance of each feature within the RF. To improve the performance of the model, the variables with the lower MDGI were recursively removed until the value of the OOB prediction error measured on the training dataset reached its lowest value [41].

## Results

### Recurrence volume identification

**Fig. 3:**
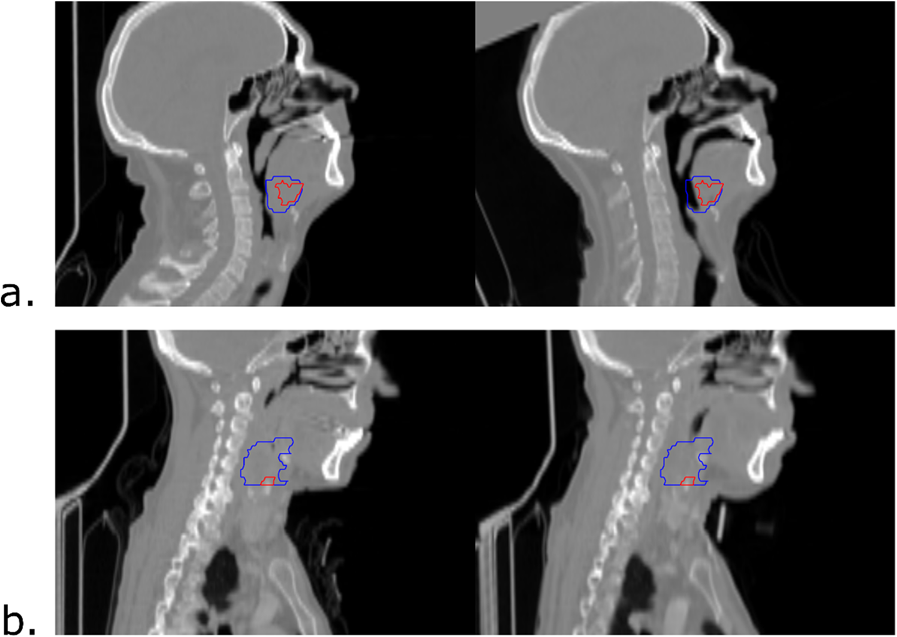
Examples of both inaccurate and accurate structures matching. Left: pre-treatment CT. Right: post-treatment CT. **a** Inaccurate structures matching. Although the skull, the jaw, and the top of the spinal cord are aligned, the tongue of the patient appears in a different position between the two scans, leading to a misalignment of pertinent structures for tumor recurrence analysis. **b** Accurate structures matching. The skull, jaw and spinal cord are aligned and no anatomical differences between the soft tissues were found nearby the tumor. Blue: pre-treatment tumor. Red: post-treatment tumor

The mean dice score measured on bones segmentations in the ROI used to perform the registration was 0.70 ± 0.14. After a qualitative assessment, 15 pairwise registrations out of 26 proved to be anatomically accurate enough to identify and characterize recurrence volume. Among these patients, tumor localization was distributed as oropharynx for six patients, hypopharynx for four patients, oral cavity for three patients, and larynx for two patients. Examples of accurate and inaccurate structures matching are presented in Fig. 3. The Fleiss’s Kappa for inter-observer agreement was 0.66.

The inter-rater reliability, computed using the Fleiss's Kappa for inter-observer agreement was 0.66. Within the 15 accurate identifications, recurrence volume represented on average 23% of the GTV (standard deviation =12%).

### Recurrence and responder volume characterization

Table 2 presents the histogram-based features computed within both GTVresponder and GTVfailure. The minimum SUV (SUVmin) and the mean SUV (SUVmean) were significantly different between GTVfailure and GTVresponder.

**Table 2:** Mean histogram feature comparison between GTVfailure and GTVresponder, with associated Wilcoxon signed-rank test *p* values

Histogram Features	GTVfailure	GTVresponder	*p* values
Minimum	2.79 ± 2.23	1.21 ± 0.38	0.031*
Maximum	14.53 ± 5.87	14.93 ± 6.68	0.747
Mean	7.37 ± 3.47	5.40 ± 1.90	0.007*
Variance	11.57 ± 11.28	13.71 ± 14.84	0.459
Skewness	0.13 ± 0.32	0.07 ± 0.10	0.517
Kurtosis	0.46 ± 0.95	0.28 ± 0.51	0.517

*GTVfailure:* recurrence volume within the primary tumor, *GTVresponder:* responder volume within the primary tumor

* Significant difference (*p* ≤ 0.05) between GTVfailure and GTVresponder

**Fig. 4:**
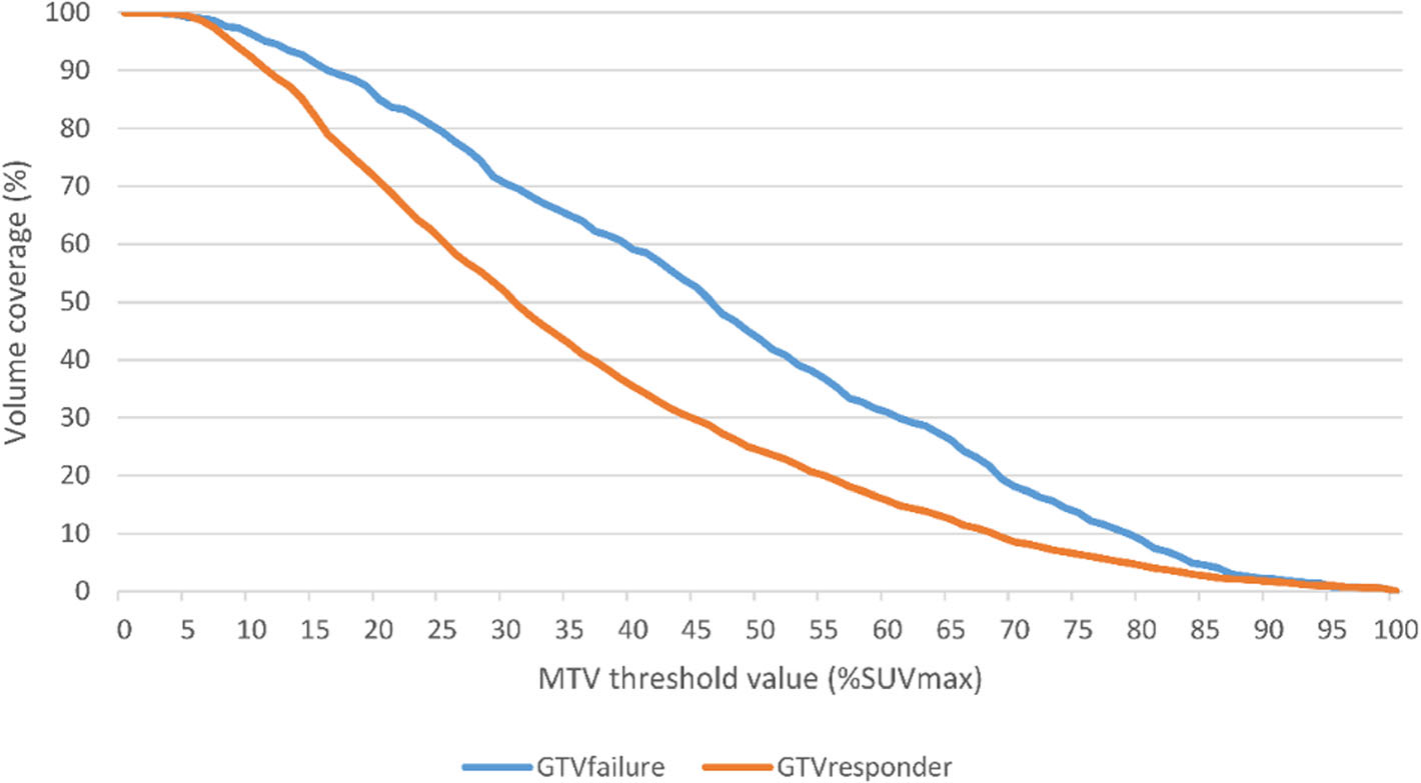
Percentage of GTVfailure (red line) and GTVresponder (blue line) covered by the MTV as a function of % SUVmax. The MTV covers a higher percentage of GTVfailure than GTVresponder for threshold values ranging from 15% to 80%. However, no threshold value allowed to cover most of GTVfailure without covering most of GTVresponder. *GTVfailure* recurrence volume within the primary tumor, *GTVresponder* responder volume within the primary tumor, *MTV* metabolic tumor volume, *SUVmax* maximum standard uptake value

The MTV covers a higher percentage of GTVfailure than GTVresponder (Fig. 4). The MTV_50+10_ covered on average 89.8% (range 48.6–100) of the recurrence volume and 96.9% (range 82.1–100) of the pre-treatment GTV.

### Voxel-wise prediction of the recurrence volume

**Fig. 5:**
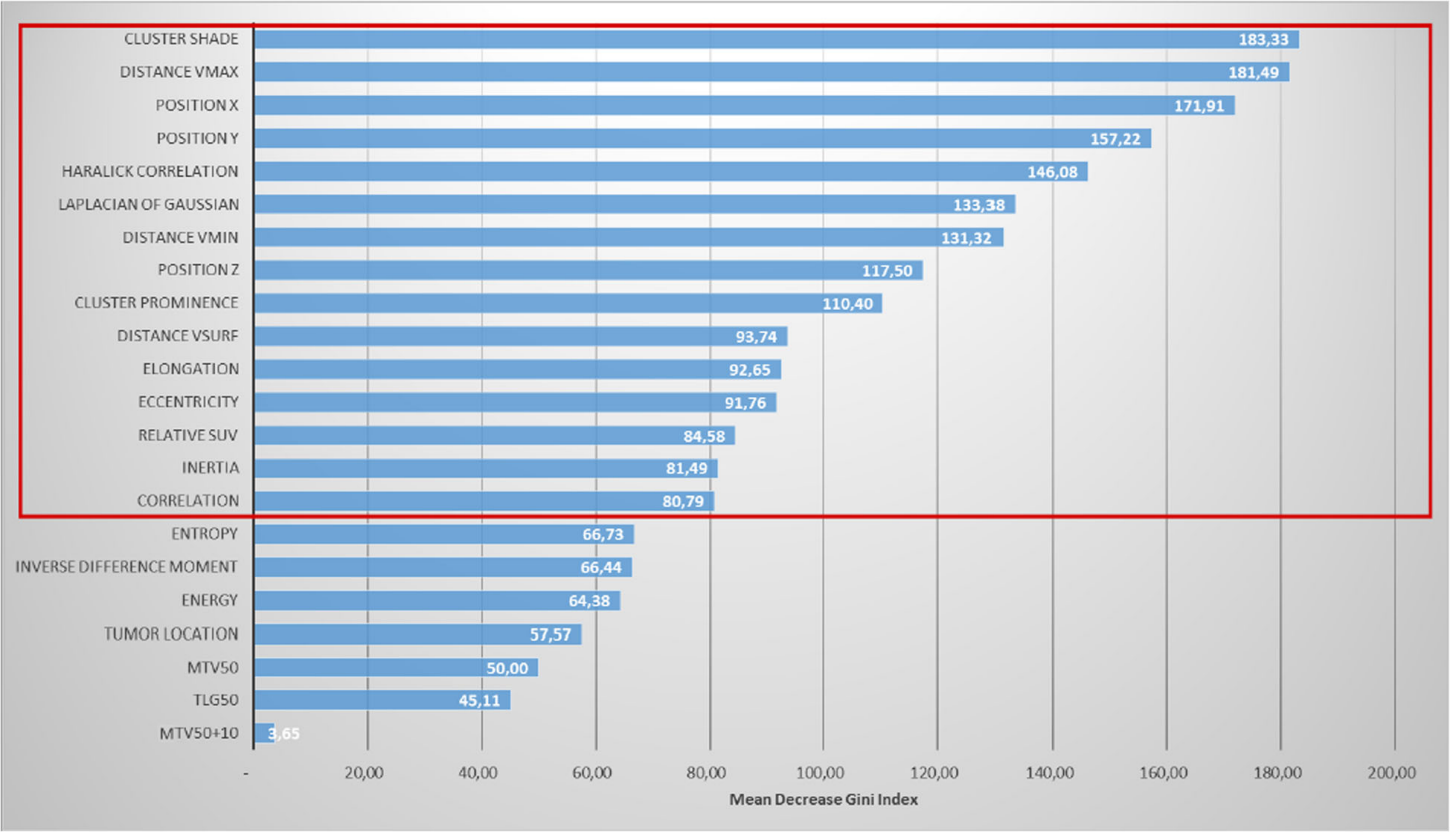
Mean Decreasing Gini Index (MDGI) of features used to train the random forest (RF). The most important variables in the RF have the higher MDGI value, here, the cluster shade, the distance to vmax, the spatial position (x,y), Haralick Correlation. etc. The red box indicates the features kept to train the RF after the recursive suppression of the less important features

Figure 5 presents the mean importance of features within the leave-one-out RF models, according to the MDGI.

**Fig. 6:**
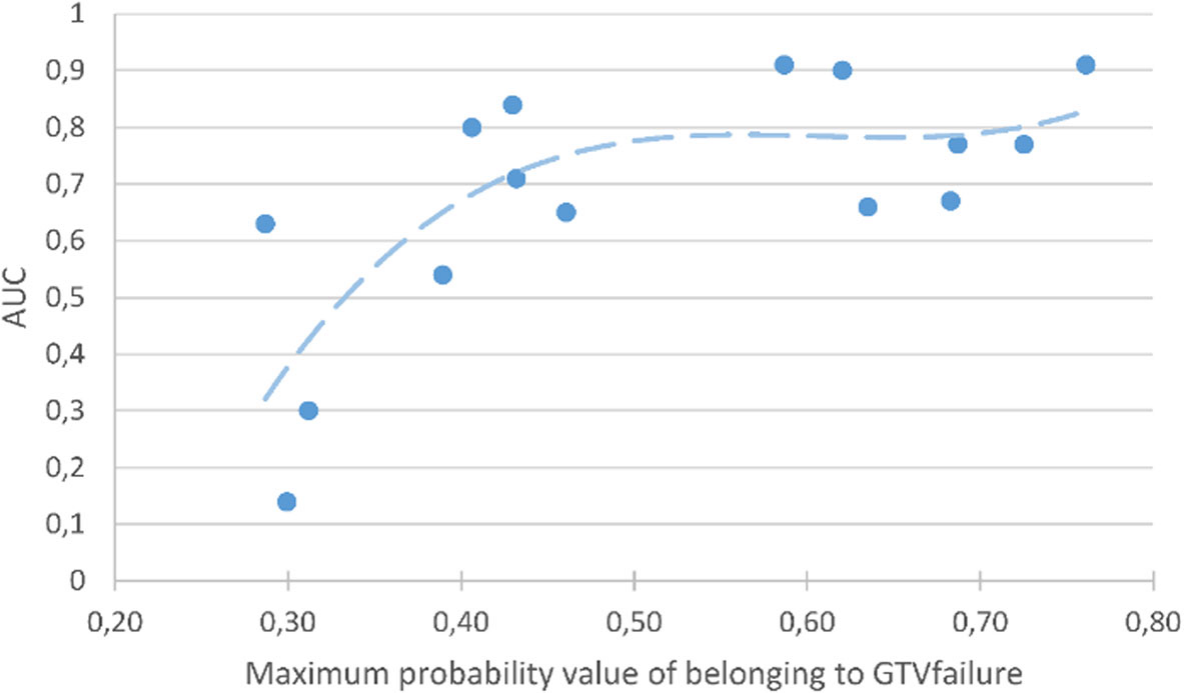
Area under the curve (AUC) of probability maps plotted against their corresponding maximum probability value of belonging to GTVfailure. A strong correlation between the AUC and the value of the voxel with the maximum probability of belonging to GTVfailure was found across the predictions performed in this study, suggesting that the reliability of the prediction can be assessed using the maximum probability value of belonging to GTVfailure

**Fig. 7:**
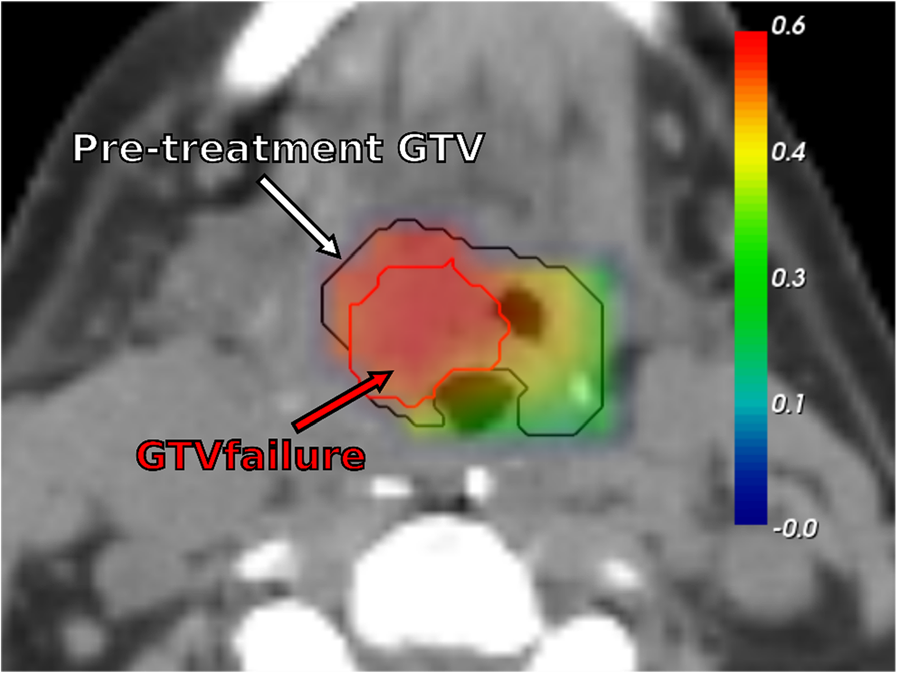
Example of probability map of recurrence, generated with random forest, overlaid on the pre-treatment CT. Black contour: pre-treatment GTV. Red contour: GTVfailure

Fifteen features out of 22 were kept as relevant to train the RF after recursive suppression of the less important features. Mean OOB prediction error on the training dataset was 0.041 (median = 0.040, range 0.039–0.045), and the mean AUC on the testing dataset was 0.68 (median = 0.71, range 0.14–0.91). Fig. 6 shows that a strong correlation (*r* = 0.64) exists between the value of the voxel with the maximum probability of belonging to GTVfailure and the AUC of the corresponding probability map. Fig. 7 presents an example of probability map generated by the model trained with the 15 retained features.

## Discussion

To our knowledge, our study is the first to locally compute the probability of a voxel to belong to a recurrence region within the GTV. This study was based on voxel-wise features extracted from pre-treatment PET/CT. The prediction model was validated using a leave-one-out scheme. A spatial characterization allowed to describe sub-regions likely responsible of recurrence origin which may be then used to guide dose tumor painting. In this manner, the obtained model provides a way forward to predict recurrence regions by only using the pre-treatment PET/CT.

A customized rigid co-registration method was implemented to identify recurrence volume. 3D Euclidean distances were computed on bone segmentations helping to steer the registration algorithm and allowing to account for changes in soft tissues anatomies due to the CRT treatment. The performance of the block-matching algorithm was increased compared to CT intensity based registration of regions with similar intensities [31].

The average dice score obtained on bone segmentations after registration was low. This may be explained by the changes in patients’ position between two image acquisitions. The dice score is sensitive to the size of the defined ROI, which is different for every patient in the current study and might be too small in certain cases to obtain high dice score values. However, the aim of the registration was to identify the origin of recurrence in pre-treatment scans. Thus, the quality of this identification cannot be measured using similarity metrics and the value of the dice score could not be used to validate registration results in the current study.

Currently, the gold standard method to identify head and neck cancer recurrence origin is to propagate the center of mass of the post-treatment tumor to the pre-treatment scan using non-rigid registration methods. GTVfailure has been defined by adding a 4 mm radius margin to this center of mass, to account for registration and delineation uncertainties. Although, this procedure allows the identification of a global recurrence origin even in the case of important anatomy changes between scans, the identification of GTVfailure is not accurate enough to perform a voxel-wise analysis.

Thus, we privileged the use of a rigid registration method for preserving anatomical correspondences despite intra-patient changes and to select patients with accurate identification of GTVfailure. The accuracy of recurrence volume identification was manually assessed by a senior radiation oncologist. Eleven patients were therefore excluded from the study considering that rigid registration did not allow to identify the recurrence volume with enough accuracy, in the cases where the anatomy of the subject was different between the pre- and post-treatment PET/CT.

A regional characterization of FDG-uptake features showed that the mean FDG-uptake (SUVmean) was significantly higher in GTVfailure than in GTVresponder. However, the recurrence volume was not entirely covered by MTV_50_ and the SUVmax was not a predictor of recurrence volume location, suggesting that histogram-based FDG-uptake features are not the best suited to guide dose-escalation strategies. These results are in line with Mohamed et al. [24], who suggested to add a margin of 10 mm to the MTV_50_ to cover the majority of recurrences. Nonetheless, the MTV_50+10_ also covers most of the GTV. Thus, this feature also limits the use of dose escalation strategies as it is not able to provide a precise identification of the radio-resistant regions likely to trigger recurrence.

Regional FDG-uptake features appear as inaccurate predictors of recurrence volume. As opposed to regional approaches, we performed voxel-wise analysis of FDG-uptake using radiomics features via a random forest machine learning strategy. Thus, each voxel becomes informative and relevant to build a relapse region by its progressive aggregation when they are likely recurrent. The use of radiomics features was of particular interest as it allowed to extract both global and local information on the spatial relations between FDG-uptakes computed on small volumes, in addition to the FDG-uptake information itself [27, 42]. Moreover, textural analysis provides prognostic information on pre-treatment FDG PET/CT in HNSCC [43]. It should be noted that the parameters used to compute radiomics in this study were chosen in agreement with the Image Biomarker Standardization Initiative [34].

The predictive capability of each feature was assessed using a leave-one-out cross-validation RF, with MDGI computation. The ranking of features using the MDGI showed that radiomics and voxel location features were the most predictive features of recurrence volume. The first feature directly linked with histogram-based FDG-uptake is the relative SUV, ranked as 13th according to the MDGI. The feature importance ranking also showed that the inclusion of voxels within the MTV_50+10_ volume is not relevant for recurrence volume prediction at the voxel level. This was expected as the MTV_50+10_ covers a large area of the GTV and is rather a global characteristic.

A backward feature selection strategy was used to improve the prediction of the model. However, the model obtained did not provide acceptable results for a clinical use. A strong correlation was found between the value of the voxel with the maximal probability of belonging to GTVfailure and the AUC of the corresponding probability map, suggesting that the maximal probability of belonging to GTVfailure can be used to assess the prediction reliability of the model.

Our study exhibits some limitations. The accuracy of the registration was qualitatively assessed by a single expert. Inter-observer variability can however arise, impacting the reproducibility of the experiments. A potential weakness of this approach is the risk of selection of a particular sub-tumor type (i.e., without major anatomical variations), or particular radioresistant tumor (as suggested by the median time to recurrence). Further validation of our methodology may require multiple readers at different centers, and an evaluation of the agreement between different experts to identify adequate registrations.

After the registration step, a significant number of patients were excluded from the study to ensure an accurate identification of the recurrence volume. Therefore, the prediction of recurrence volume was performed on 15 patients only. However, the training of the RF model was performed at the scale of the voxel, which means that a very high number of samples was used to generate a robust model. Each voxel is a different entity so independently considered, allowing their aggregation in regions which are predictive for the risk of recurrence even with a small number of patients. All patients were scanned using the same PET machine. Then, the model should be also customized for other PET/CT protocol acquisitions.

Due to the limited number of patients, no external validation was performed. Internal validation by leave-one-out already showed a reliable performance, suggesting that a voxel-wise machine learning approach, trained with the appropriate features and number of patients, may help identifying the location of radio-resistant regions within the pre-treatment GTV to guide dose escalation strategies. FDG PET has a poor resolution at the voxel level, which can complicate the analysis. However, the correlation between the maximal probability of belonging to GTVfailure and the reliability of the prediction provided by the model would help to determine a sub-region of high risk of recurrence.

Textural analysis was extracted from GTV, which was manually defined by a radiation oncologist. Due to the impact of the segmentation method on the textural analysis [43], there is a risk of variability in applying the model in another center. However, several studies did not find significant difference in inter-observer GTV delineation of head and neck cancers [44, 45].

The algorithm was not obviously evaluated on tumors that do not fail therapy. However, our model aims to identify tumor sub-regions where recurrence is likely to occur, rather than predicting the risk of recurrence. It is possible that the algorithm might identify regions at risk for recurrence in tumors that would be adequately treated with standard therapy. We therefore propose that a prognostic model [23, 43] be used first to identify tumors at risk for local recurrence prior to applying our methodology.

## Conclusion

Voxel-wise analysis based on both radiomics and spatial location within the tumor seems to be very promising to identify recurrence origin. The recurrence was not correlated with FDG-uptake alone, which raises concern on the suitability of using dose tumor painting by numbers. However, a voxel-based prediction of recurrence volume may be of interest to develop instead a new approach of dose tumor painting by recurrence risk. Supplementary data are nevertheless required to confirm the potential of the approach presented in this study to predict the recurrence volume.

## Data Availability

The datasets generated and/or analyzed during the current study are available from the corresponding author on reasonable request.
